# A Mutation in Plant-Specific SWI2/SNF2-Like Chromatin-Remodeling Proteins, DRD1 and DDM1, Delays Leaf Senescence in *Arabidopsis thaliana*

**DOI:** 10.1371/journal.pone.0146826

**Published:** 2016-01-11

**Authors:** Eun Ju Cho, Seung Hee Choi, Ji Hong Kim, Ji Eun Kim, Min Hee Lee, Byung Yeoup Chung, Hye Ryun Woo, Jin-Hong Kim

**Affiliations:** 1 Advanced Radiation Technology Institute, Korea Atomic Energy ResearchInstitute, 29 Geumgu-gil, Jeongeup-si, Jeollabuk-do, 56212, Republic of Korea; 2 Department of New Biology, DGIST, Daegu, 42988, Republic of Korea; 3 Department of Radiation Biotechnology and Applied Radioisotope Science, University of Science and Technology, 217 Gajeong-ro, Yuseong-gu, Daejeon, 34113, Republic of Korea; Universidad Miguel Hernández de Elche, SPAIN

## Abstract

Leaf senescence is a finely regulated complex process; however, evidence for the involvement of epigenetic processes in the regulation of leaf senescence is still fragmentary. Therefore, we chose to examine the functions of DRD1, a SWI2/SNF2 chromatin remodeling protein, in epigenetic regulation of leaf senescence, particularly because *drd1-6* mutants exhibited a delayed leaf senescence phenotype. Photosynthetic parameters such as Fv/Fm and ETRmax were decreased in WT leaves compared to leaves of *drd1-6* mutants after dark treatment. The WT leaves remarkably lost more chlorophyll and protein content during dark-induced senescence (DIS) than the *drd1-6* leaves did. The induction of senescence-associated genes was noticeably inhibited in the *drd1-6* mutant after 5-d of DIS. We compared changes in epigenetic regulation during DIS via quantitative expression analysis of 180-bp centromeric (*CEN*) and transcriptionally silent information (*TSI*) repeats. Their expression levels significantly increased in both the WT and the *drd1-6* mutant, but did much less in the latter. Moreover, the delayed leaf senescence was observed in *ddm1-2* mutants as well as the *drd1-6*, but not in *drd1-p* mutants. These data suggest that SWI2/SNF2 chromatin remodeling proteins such as DRD1 and DDM1 may influence leaf senescence possibly via epigenetic regulation.

## Introduction

Leaf senescence is an endogenously controlled degenerative process that occurs in the final stage of leaf development, leading to leaf death [1, 2]. During leaf senescence, a series of events involving the degradation of chloroplast, decrease in photosynthetic activity, loss of chlorophyll, and the recycling of valuable nutrients to other parts of the plant, occur [3]. Initial chloroplast degradation is followed by nuclear and vacuolar breakdown. This latter mechanism is accompanied by the release of nucleases and proteases, acidification of the cytoplasm, and rapid degradation of nucleic acids and proteins [4, 5]. Developmental senescence is a highly regulated genetic process that occurs in an age-dependent manner [6]; however, it is also influenced by complex interaction of developmental stage with various internal and external factors [2]. Internal factors include developmental age, diverse phytohormones, and reproductive development. External or environmental factors include stresses such as high light intensity, temperature extremes, drought, ozone, shading, nutrient deficiency, and pathogen infection. It is clear that multiple pathways regulating multiple internal and external factors exist and are interconnected to form a complex network that regulates senescence.

Studies of changes in gene expression by using microarray analysis have shown that senescence is often regulated by transcription factors. Over 800 genes have been identified as senescence-associated genes (SAGs), and among them, over 100 genes encode transcription factors [6, 7], including WRKY, NAC, MYB, TUB, bZIP, and C2H2 transcription factor families. The *Arabidopsis WRKY53* transcription factor plays a central role in the regulation of the early stages of senescence [8–11]. A knock-out line of the *WRKY53* gene shows delayed leaf senescence; on the other hand, overexpression of this gene leads to precocious senescence [9, 11]. The WRKY53 transcription factor controls several SAGs involved in the control of leaf senescence [9].

Epigenetic regulation plays an important role in cellular senescence and organism aging in higher organisms [11–13]. The epigenetic mechanisms including DNA methylation, histone modification, and ATP-dependent chromatin remodeling control expression of the senescence-associated genes (*SAGs*) by reprogramming of chromatin state with aging. Interestingly, global DNA hypomethylation and specific loci hypermethylation occur in aging. Recently, many molecules that control global alteration in chromatin structure during senescence have been analyzed. Screening of activation-tagged lines in *Arabidopsis* for delayed leaf senescence identified the *ORE7/ESC* gene, which encodes an AT-hook DNA-binding protein. Expression of *ORE7* results in a dosage-dependent effect on the initiation of leaf senescence and interphase chromatin organization [14]. It was also reported that histones at the WRKY53 promoter and coding regions undergo H3K4 methylation to be active chromatin state for upregulation of target SAGs after onset of leaf senescence [11]. However, in case of the SWITCH/ SUCROSE NONFERMENTING (SWI/SNF) chromatin remodelers, its role in leaf senescence has not been explored in spite of existence of a number of SWI/SNF chromatin remodelers. The SWI/SNF chromatin remodelers can alter chromatin structure through ATP hydrolysis and play important roles in diverse developmental process. DRD1 (AGI locus no. At2g16390) is a putative chromatin remodeling protein and member of the plant-specific subfamily of SWI2/SNF2-like proteins [15, 16]. DRD1 is a well-known epigenetic regulator, which was the first SNF2-like protein implicated in RNA-guided epigenetic modification of other genes [15]. It also cooperates with PolVb (NRBD1b and NRBD2a) to facilitate RNA-directed DNA methylation (RdDM) and silencing of homologous DNA [17]. A genetic screen has also revealed that RNA-directed non-CG methylation requires DRD1 [18].

In this study, we examined senescence symptoms in the *Arabidopsis drd1-6* mutant at the physiological and molecular level. We found significantly delayed leaf senescence in rosette leaves and whole plant in *drd1-6* mutant during DIS and natural senescence. Similar phenotypes were observed in *ddm1-2* mutant as well as *drd1-6* mutant, but not in *drd1-p* mutant. Identification and analysis of gene expression by microarray in *drd1-6* mutants during DIS indicated that genes were clustered according to their responsiveness to dark treatment and gene expression patterns. In addition, using quantitative gene expression analysis of 180-bp centromeric (*CEN*) and transcriptionally silent information (*TSI*) repeats, we showed that epigenetic regulation occurs during DIS. This study suggests that a SWI2/SNF2-like protein, DRD1, is positively associated with leaf senescence possibly via epigenetic regulation.

## Materials and Methods

### Plant material and growth conditions

**Fig 1:**
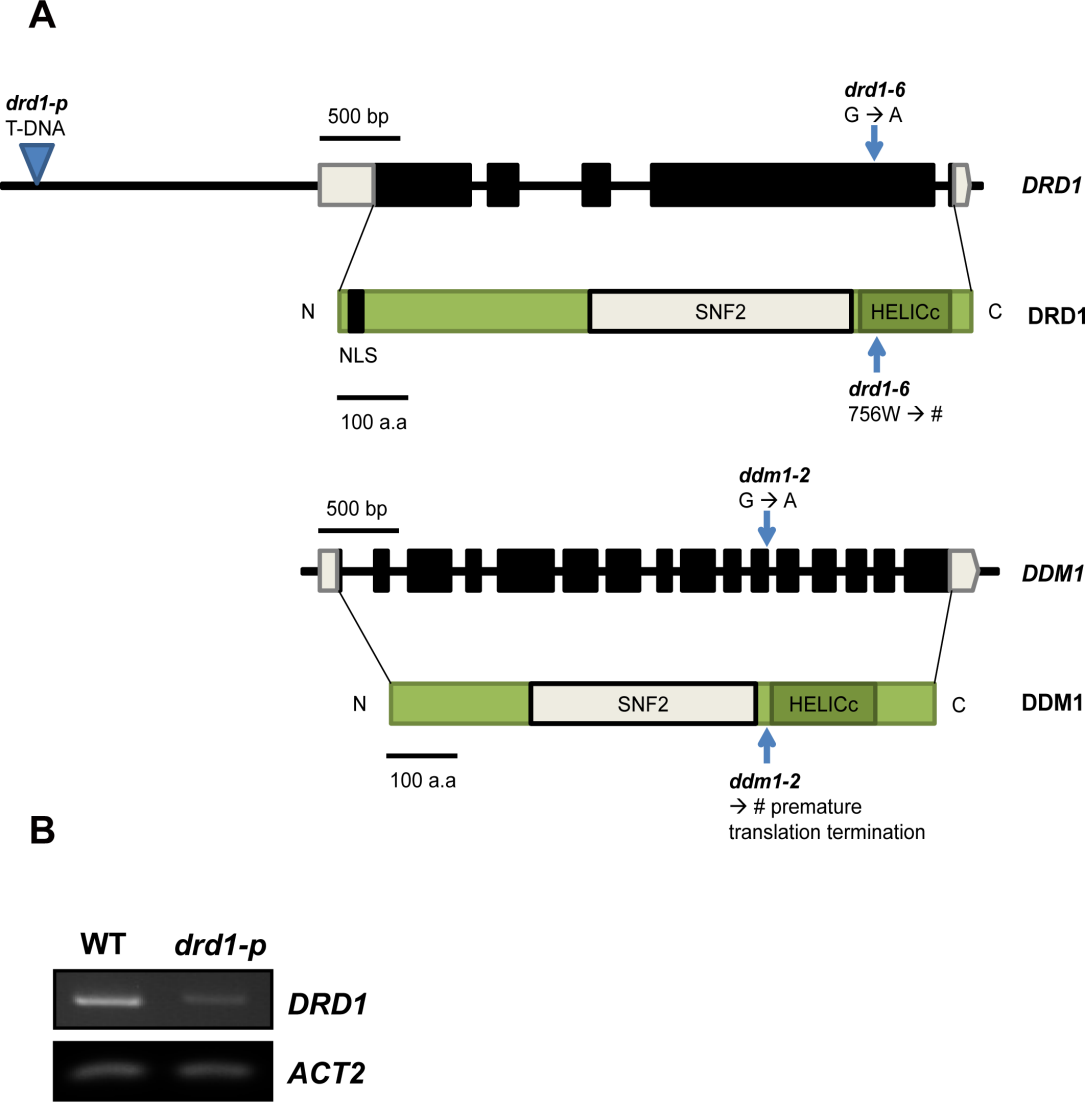
The positions of mutations and expression of *DRD1* gene. (A) Domain structures of DRD1 and DDM1 and positions of *drd1-6*, *drd1-p* and *ddm1-2*. Amino acid sequence change from tryptophan (W) to stop codon in helicase superfamily C-terminal (HELICc) domain in *drd1-6*. The triangle indicates the position of T-DNA insertion in *drd1-p* mutant. In case of *ddm1-2*, substitution of G to A in the splice donor site of intron 11 brings about lack of helicase superfamily C-terminal (HELICc) domain. (B) RT-PCR analysis of *DRD1* and control *ACTIN2* genes in WT and *drd1-p* mutant leaves. The *drd1-p* mutant displayed a decrease in *DRD1* expression levels compared to WT.

*Arabidopsis thaliana* ecotype Columbia (Col-0) wild type (WT) and *drd1-6* mutant seeds were sterilized in 70% (v/v) ethanol for 1 min, and suspended in 30% Clorox, 0.01% Triton X-100 for 15 min, rinsed with sterile deionized water. Sterilized seeds were stratified at 4°C for 2 day and then grown on the soil in a growth chamber at 100 ~ 130 μmol m^-2^ s^-1^ and a 16 h photoperiod at 22/18°C (Day/Night). *Arabidopsis drd1-6* allele used in this study has a nucleotide sequence change from G to A, which results in an amino acid substitution of the 756^th^ tryptophan (W) to a stop codon in helicase superfamily C-terminal (HELICc) domain (Fig 1A). The *drd1-p* allele that carries a T-DNA insertion in the promoter of *DRD1* was obtained from the Salk Collection (SALK_132061) and was confirmed as a knockdown mutant by a PCR-based method (Fig 1B). In case of *ddm1-2*, substitution of G to A in the splice donor site of intron 11 brings about a deletion, a frameshift, and a premature translation termination, resulting in lack of HELICc domain (Fig 1A). The *drd1-6* and *ddm1-2* mutants have been reported previously [15, 19].

### Senescence analysis

For the dark-induced senescence (DIS) assay, rosette leaves from 28-day-old *Arabidopsis* WT and *drd1-6* mutant plants were excised and placed on moisturized filter papers in petri dishes. The plates were kept under darkness for 7 days. The rosette leaves of 28-day-old plants were used for treatments of individually darkened leaves (IDL). IDL treatments were performed as described previously [20]. The rosette leaves were covered with aluminum foil for 5 days, and then harvested.

### RNA isolation and expression analysis

Total RNA was isolated from rosette leaves of *Arabidopsis* using RNeasy Mini Kit (Qiagen, Hilden, Germany) according to manufacturer’s instructions. For RT-PCR, the first strand cDNA was produced by Maxime RT PreMix Kit (iNtRON Biotechnology, Seongnam, Korea), and then used in PCR with Maxime PCR PreMix Kit (iNtRON Biotechnology, Seongnam, Korea). The gene-specific primers described in S1 Table were used for PCR reactions. *ACTIN2* gene was used as a control. PCR conditions were as follows: template DNA was denatured at 94°C for 2 min, and then amplified by 30 cycles at 94°C for 20 sec, 55°C for 10 sec, 72°C for 30 sec, and extended at 72°C for 5 min. Quantitative real-time PCR was performed in the 7300 Real-Time PCR system (Applied Biosystems, Massachusetts, USA) using SYBR Premix Ex Taq (Takara Bio Inc., Shiga, Japan). The relative expression ratios of target genes were calculated in comparison with a reference gene, *ACTIN2*, using the comparative C_T_ method [21].

### Measurement of chlorophyll and protein content

Chlorophyll and protein were extracted from rosette leaves of WT and *drd1-6* mutant and quantified as described previously [22]. One hundred mg of leaf tissues were ground with liquid nitrogen, and then 1 ml 100% (v/v) acetone was added. After centrifugation at 10,000 x g for 10 min at 4^°^C, supernatants were harvested and their absorbance measured at two wavelengths: A645, and A662. The chlorophyll level was calculated as follows: C_a+b_ = 18.09 x A645 + 7.05 x A662. Unit was converted from μg Chl/ml to μg Chl/g fresh weight. Total protein was extracted by grinding leaf tissues in protein extraction buffer (50 mM Tris, pH 8.0, 0.25 M sucrose, 2 mM DTT, 2 mM EDTA, 1 mM PMSF, and 1x Complete protease inhibitor cocktail [Roche, Mannheim, Germany]). The protein content was determined after centrifugation at 10,000 x g for 10 min at 4^°^C by the Bradford method [23].

### Photosynthesis analysis

For evaluation of photosynthetic activity, chlorophyll fluorescence was measured using the IMAGING-PAM chlorophyll fluorometer (WALZ, Effeltrich, Germany) as described previously [24]. The maximum photochemical efficiency (Fv/Fm) of PSII was deduced from chlorophyll fluorescence. The maximal electron transport rate (ETRmax) of WT and the *drd1-6* mutant was obtained from the relative ETR vs. photosynthetic photon flux density (PPFD) curve.

### Blue-native PAGE and immunoblot analysis

Blue-native PAGE were followed the experimental procedures as described previously [25] with some modifications. Thylakoid membranes were isolated from DIS-treated rosette leaves in WT and *drd1-6* mutant and solubilized with 1% (w/v) n-dodecyl-β-D-maltoside and loaded to 6 ~ 12% gradient gel. For immunoblot analysis, thylakoid membrane proteins were denatured with 100 mM Tris-HCl, pH6.8, containing 17% (v/v) glycerol, 3.5% (w/v) SDS, 6 M urea, and 10% (W/V) β-mercaptoethanol for 30 min at 70°C. They were separated via SDS-Urea PAGE (12% polyacrylamide) and were detected with rabbit antiserum raised against the DE loop (Residues 225 to 249) of the spinach D1 protein as described previously [26].

### Affymetrix ATH1 Genome Array and data analysis

Genome-wide gene expression analysis was conducted using the GeneChip® *Arabidopsis* ATH1 Genome Array (Affymetrix, Santa Clara, CA). All experimental procedures such as RNA preparation, RNA quality check, conversion of RNA into double-stranded cDNA, generation of biotin-labeled cRNA from the double-stranded cDNA, and hybridization of the biotin-labeled cRNA with the ATH1 Genome Array, were performed according to the instructions and recommendations provided by Affymetrix. Following the hybridization, the GeneChip arrays were scanned using a GeneChip® Scanner 3000 7G (Affymetrix, Santa Clara, CA) and the scanned data were analyzed for differentially expressed genes (DEG) of control vs. test samples via a DAVID program (http://david.abcc.ncifcrf.gov/). Gene ontology analysis was performed using the DEGs with more than two-fold change between control and test samples from two independent experiments.

## Results

### The *drd1-6* plant exhibits delayed leaf senescence

**Fig 2:**
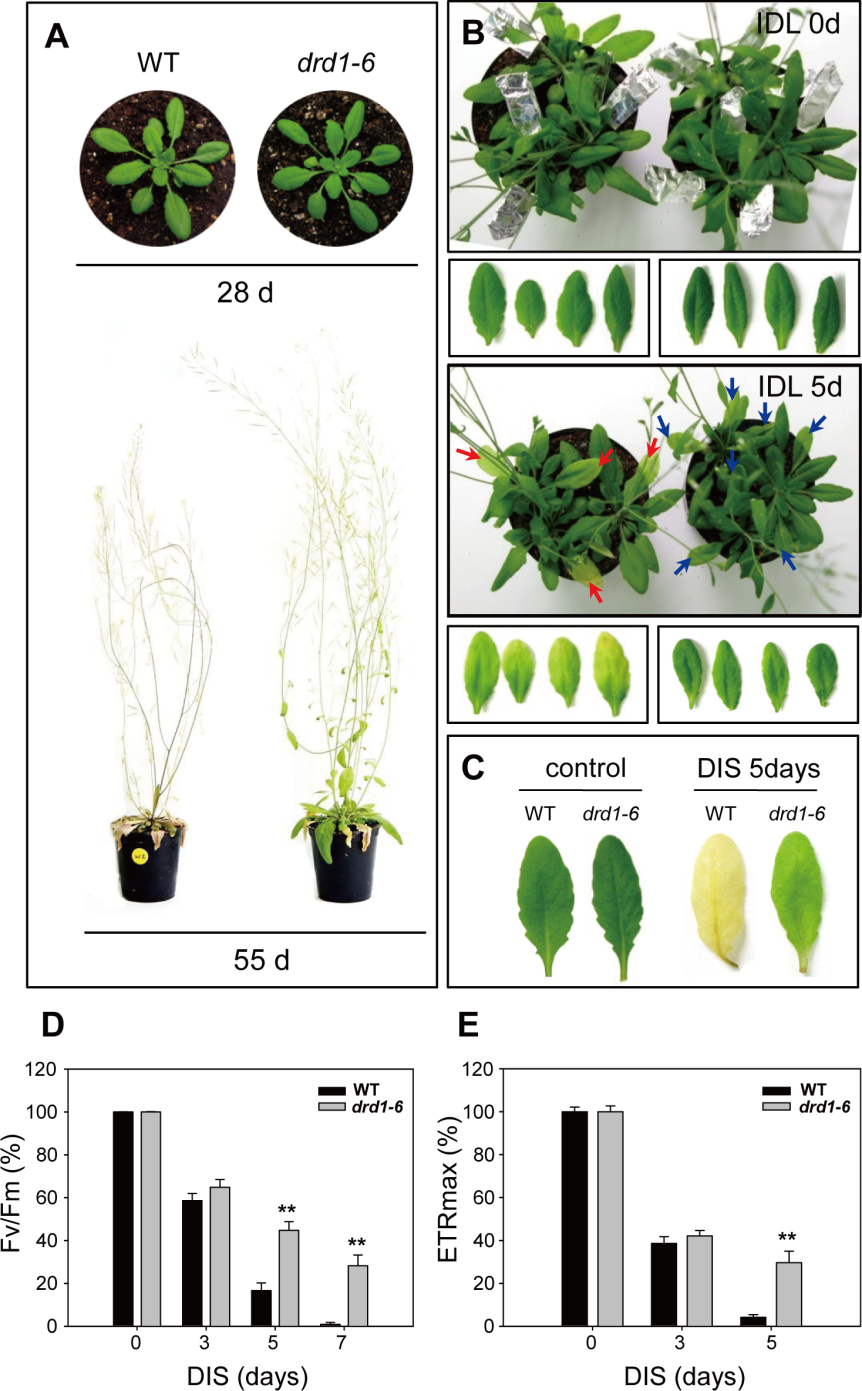
Delayed leaf senescence symptoms in the *drd1-6* mutant. (A) Phenotypes of 28-day-old and 55-day-old wild-type (WT) and *drd1-6* mutant whole plants. (B) Individually darkened leaf (IDL) senescence of WT (left) and *drd1-6* (right) plants. Rosette leaves of 28-day-old WT and *drd1-6* mutant (IDL 0 d) were induced to undergo senescence for 5 d under dark conditions (IDL 5 d). The red and blue arrows indicate 5 d IDL of WT and *drd1-6* plants, respectively. (C) Phenotypes of detached WT and *drd1-6* leaves after 5-d dark incubation. (D) Photochemical efficiency of photosystem II (Fv/Fm) and (E) maximal electron transport rate (ETRmax) in WT and the *drd1-6* leaves were examined at the indicated days during dark-induced senescence (DIS). Data represent average values ± SE (n = 27) of three independent experiments. * indicates *P* < 0.01 by student’s t-test.

We observed strong delay of age-dependent natural senescence and extended life span of whole plant in the *drd1-6* mutant. DRD1 is a representative member of SWI2/SNF2 subfamily and is required for either Pol V transcription or activation in RdDM pathway. To elucidate a relationship between senescence and SWI2/SNF2 chromatin remodelers in *Arabidopsis*, we analyzed the *drd1-6* mutant in this study. The *drd1-6* mutant had a nucleotide sequence substitution from G to A, which results in an amino acid substitution of the 756^th^ tryptophan (W) to a stop codon in the helicase superfamily C-terminal (HELICc) domain (Fig 1). To study the effect of *drd1-6* mutation on leaf senescence, we first compared the phenotypes in mutant versus wild-type leaves. The 28-day-old WT and *drd1-6* plants have a similar phenotype as shown in Fig 2. However, we observed that flowering time was delayed by 3~8 days in *drd1-6* mutants compared to WT under our growth conditions. Although it was reported that flowering time was not affected by mutation of *DRD1* gene [27], it may be variable depending on growth conditions. In addition, while the 55-day-old WT leaves had turned completely yellow and showed signs of death, the *drd1-6* mutant leaves remained green, suggestive of a prolonged life span (Fig 2A). Generally, a prolonged observation is needed to follow the series of events in natural senescence, particularly because senescence-associated processes tend to become chaotic over time [20]. Therefore, in order to observe leaf senescence over a short period and to derive clear conclusions from these observations, we induced senescence in individually darkened leaves (IDLs). Rosette leaves of the 28-day-old WT and *drd1-6* were covered individually with foil for the dark treatment. WT leaves turned yellow after the 5-d treatment, but the *drd1-6* leaves maintained a constant green color (Fig 2B). To examine the effect of the *drd1-6* mutation in dark-induced senescence (DIS), the 28-day-old WT and the *drd1-6* rosette leaves were detached and incubated under dark conditions. After 5-d dark incubation, WT leaves had completely turned yellow and showed signs of death (Fig 2C). By contrast, the *drd1-6* leaves remained green until 7 days of DIS (S1 Fig). These data together support that the *drd1-6* mutant shows a delay not only in dark-induced leaf senescence, but also in natural senescence.

To further characterize the delayed senescence of the *drd1-6* plants, various parameters of senescence were examined. Photosystem II (PSII) efficiency has been used to measure leaf senescence, since it declines rapidly during senescence, indicating a loss of photosynthetic capabilities and ultimately death of the leaves [28]. The maximum photochemical efficiency of photosystem II (variable fluorescence / maximal fluorescence, Fv/Fm = 0.815; 100%) was markedly reduced in the WT plants and slightly reduced in the *drd1-6* mutants (Fig 2D). Another parameter—maximal electron transfer rate (ETRmax)—can also be used to measure potential photosynthetic capacity. The WT plants showed a marked decrease in ETRmax during DIS, while the *drd1-6* mutants showed a significantly lower decrease (Fig 2E). These results indicate that the *drd1-6* mutant shows considerable delay in several of the parameters used to measure leaf senescence.

**Fig 3:**
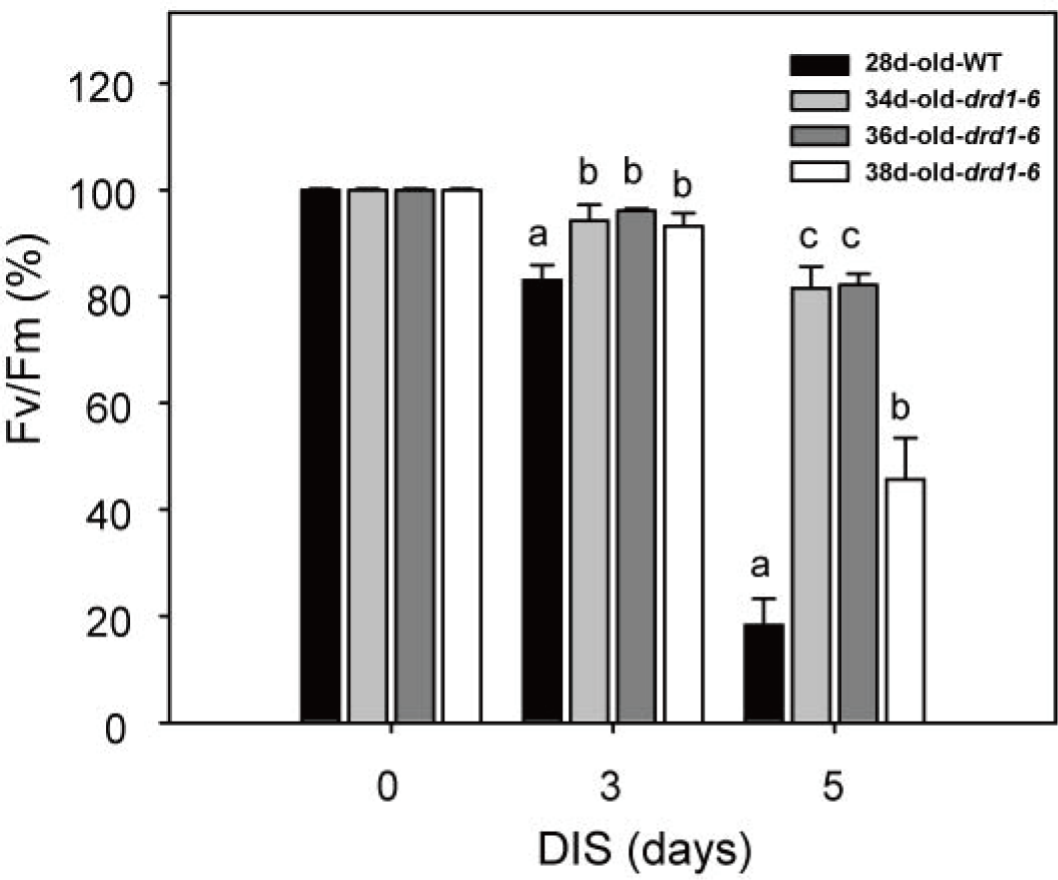
Delayed leaf senescence symptoms in the *drd1-6* mutant at later developmental stages. Rosette leaves of 28-day-old WT and the 34, 36, and 38-day-old *drd1-6* mutants were detached and darkened for 0, 3, 5 days. Photochemical efficiency of photosystem II (Fv/Fm) in WT and mutant leaves was examined at the indicated days. Data represent average values ± SE (n = 20) of independent experiments. Bars with the same letter are not significantly different at *P* < 0.05 by Tukey’s honestly significant difference (HSD) test.

Leaf senescence is affected by developmental age of plants [2]. Although we observed a strong phenotype of delayed senescence in the *drd1-6* mutant, such a phenotype might be attributed merely to a delay in plant development. To check this possibility, we compared DIS among the 28-day-old WT, 34, 36, and 38-day-old *drd1-6* plants at different developmental stages. All the *drd1-6* plants showed a much higher photochemical efficiency during DIS compared to the WT plants, supporting the delayed progress of leaf senescence (Fig 3). These results imply that the delayed leaf senescence in the *drd1-6* mutant cannot be attributed merely to a developmental difference between the WT and the *drd1-6* mutant. Accordingly, it is suggested that a mutation in DRD1 can modulate leaf senescence itself as well as plant development.

### The breakdown of photosynthetic pigments and proteins was delayed in the *drd1-6* mutant

**Fig 4:**
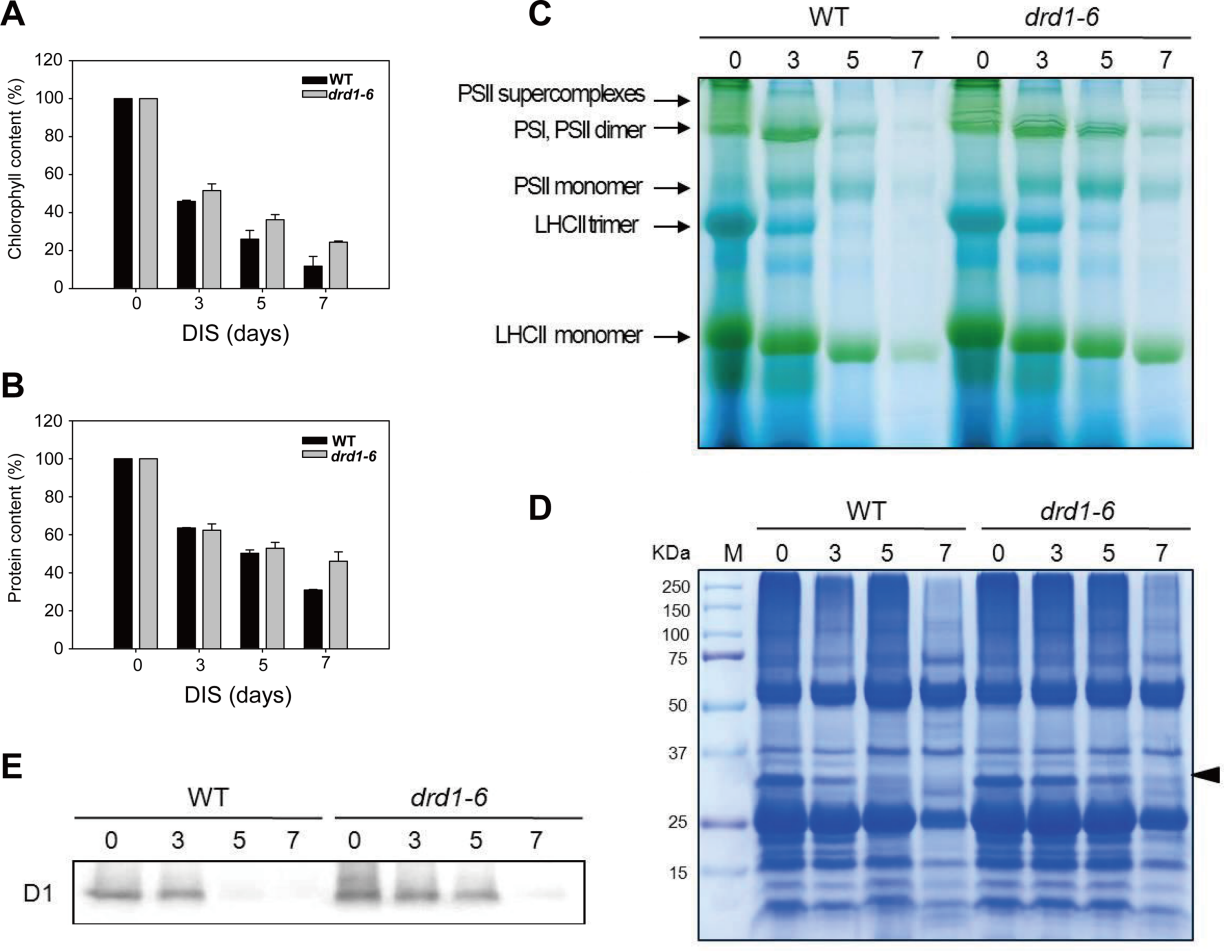
Effects on chlorophyll and protein degradation in the *drd1-6* plants during DIS. (A) Chlorophyll content was measured using rosette leaves after 0d, 3 d, 5 d, and 7 d of DIS, as indicated. (B) Protein contents from the WT and the *drd1-6* mutant leaves before (0 d) and after 3 d, 5 d, and 7 d of DIS. (C) Blue-Native Polyacrylamide Gel-Electrophoresis (BN-PAGE) of thylakoid protein complexes. Dodecylmaltoside-solubilized thylakoid membrane proteins corresponding to equal amounts of fresh weight were subjected to BN-PAGE. (D) Immunoblot analysis of thylakoid protein complexes. Thylakoid membranes were solubilized and subjected 15 μg of protein per well to SDS-PAGE. The band indicated by the arrow head was identified by LC MS/MS (see S2 Table). (E) D1 protein was identified by immunoblot analysis using anti-D1 antiserum.

As shown above, the *drd1-6* mutant exhibited delayed leaf senescence in terms of photosynthetic parameters, Fv/Fm and ETRmax. Therefore, we measured the chlorophyll and protein contents which are closely related to the photosynthetic abilities. The chlorophyll content was decreased up to 11.8% in the WT, but it was retained at a level of 24.4% in the *drd1-6* mutant after 7-d dark incubation (Fig 4A). Moreover, the protein contents of WT and *drd1-6* rosette leaves were differentially decreased up to 30.9% and 46.1%, respectively (Fig 4B and S2 Fig). When the progress of leaf senescence was assessed using chlorophyll and protein levels, the *drd1-6* mutant exhibited substantial delay in leaf senescence.

Disintegration of chloroplasts is the primary symptom of leaf senescence [2]. In the thylakoid membranes, chlorophylls are tightly bound to light-harvesting complexes (LHC) in photosystems [29]. To analyze the stability of photosystem sub-complexes during leaf senescence in detail, we isolated thylakoid membranes from the WT and the *drd1-6* leaves at 0, 3, 5, and 7-d of DIS, and then solubilized them with a mild detergent, 1% (w/v) n-dodecyl-β-D-maltoside. Blue-Native Polyacrylamide Gel-Electrophoresis (BN-PAGE) of the thylakoid membrane proteins showed increased degradation of chlorophyll-protein (CP) complexes in the WT from 3 d of DIS (Fig 4C). CP complexes were initially degraded in the order of PSII dimer to PSII monomer or LHCII trimer to LHCII monomer. Almost all the complexes were degraded at 7 d of DIS in the WT. On the other hand, the CP complexes of the *drd1-6* leaves were much less affected during the same period of DIS. Degradation of the CP complexes by DIS was delayed by approximately 2 days in the *drd1-6* mutant compared to the WT.

For further identification of the thylakoid membrane proteins primarily degraded during DIS, the solubilized thylakoid membranes were also subjected to electrophoresis on a denaturing gel. Some proteins corresponding to approximately 20~35 kDa were gradually degraded during DIS with degradation rates that were much higher in the WT than in *drd1-6* mutants (Fig 4D). We analyzed the band using liquid chromatography-coupled electrospray ionization MS/MS (LC MS/MS). We found that the band included PSII protein D1 and D2, cytochrome f, oxygen-evolving enhancer protein 1–2, chlorophyll a-b-binding protein CP26 and CP29.2, etc. (S2 Table). Immunoblot analysis revealed that D1 protein, which is approximately 32 kDa and essential for photosynthetic activity as a core component of PSII, was retained tolerably at 7 d of DIS in the *drd1-6* mutants (Fig 4E). By contrast, this protein was degraded earlier in the WT plants.

### The *DRD1* mutation causes repression of senescence-associated gene expression during DIS

**Fig 5:**
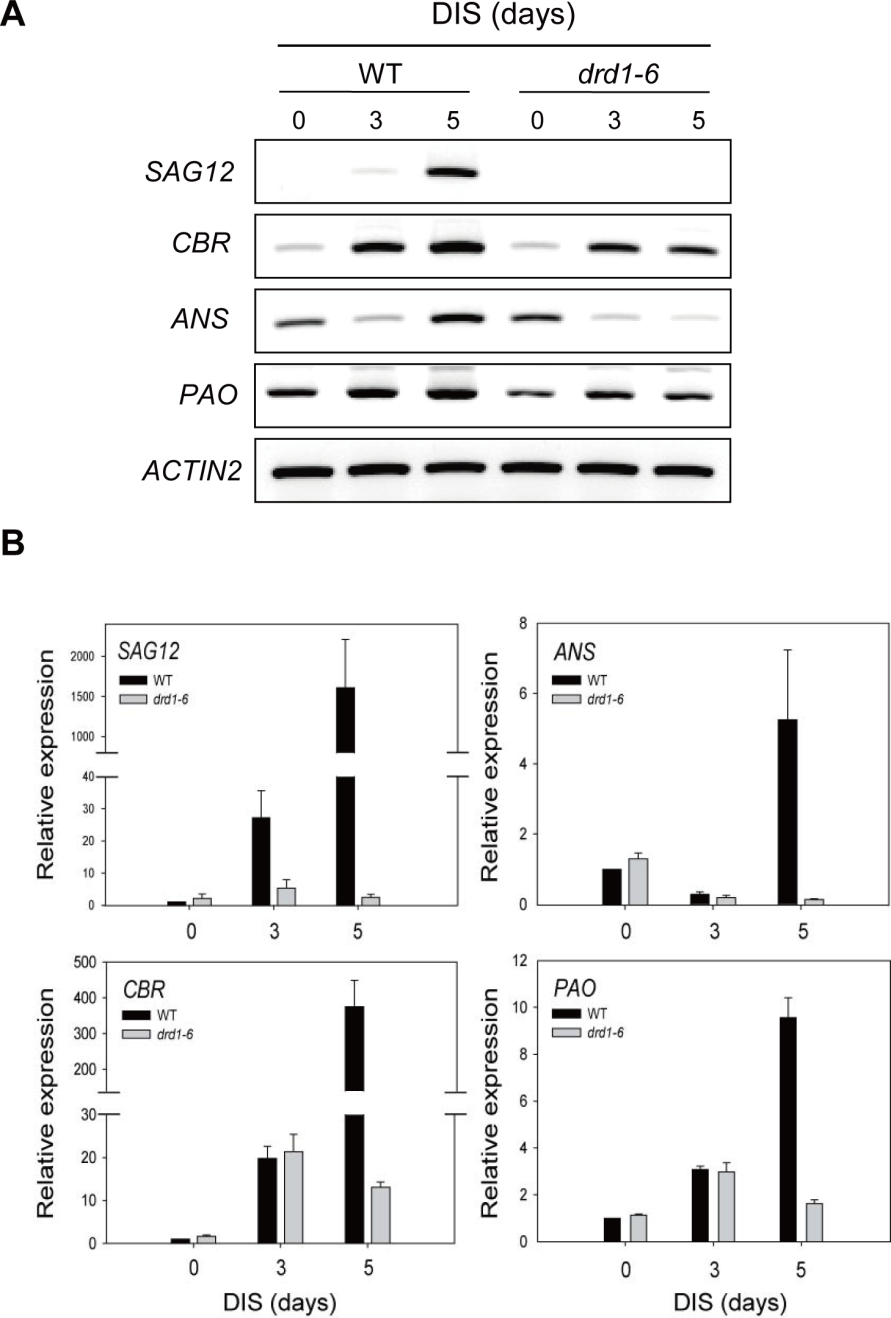
Expression of senescence-associated genes in the *drd1-6* mutant during DIS. (A) RT-PCR and (B) Quantitative real-time PCR (qRT-PCR) analysis of gene expression in WT and the *drd1-6* mutant leaves at the indicated days. *SAG12*, senescence-associated gene 12; *ANS*, antocyanidin synthase gene; *CBR*, chlorophyll b reductase gene; and *PAO*, pheophorbide α oxygenase gene. The values are normalized to *ACTIN2* expression. Data indicate the mean ± SD (n = 9) of three independent experiments.

Leaf senescence is accompanied by increased expression of senescence-associated genes (*SAGs*) [6] and decreased expression of related photosynthesis genes and protein synthesis genes (*PAGs*) [30]. To characterize the delay in leaf senescence of the *drd1-6* mutant, we analyzed the expression of various senescence-related genes by RT-PCR and qRT-PCR (Fig 5A and 5B). The expression of *SAG12*, a senescence-associated cysteine protease gene in *Arabidopsis*, was strongly enhanced in WT plants at 5 d of DIS but not significantly induced in the *drd1-6* mutants (Fig 5). Chlorophyll degradation is a key step in senescence, and a number of genes involved are under tight transcriptional control [31]. Chlorophyll degradation-related genes such as *CBR* (chlorophyll b reductase) and *PAO* (pheophorbide α oxygenase) also had higher expression levels in WT plants than in the *drd1-6* mutants during DIS (Fig 5), and this could be accountable for the differential chlorophyll contents as shown in Fig 4A. Chlorophyll was retained in the *drd1-6* mutants despite the induction of the chlorophyll degradation pathway. In contrast, the expression level of the *ANS* gene, which encodes an enzyme for anthocyanidin synthesis [32], increased in WT during DIS but decreased in the *drd1-6* mutant (Fig 5). Expression patterns of these genes obtained by RT-PCR were similar to those obtained by qRT-PCR. In senescing leaves, anthocyanins accumulate prior to chlorophyll breakdown and play a photoprotective role against the development of leaf senescence [33]. Together, these results imply that specific changes in genes that control chlorophyll and anthocyanin metabolism contribute to the green phenotype of the *drd1-6* mutants during DIS.

### Comparison of genome-wide transcriptomes in WT and *drd1-6* plants during DIS

**Fig 6:**
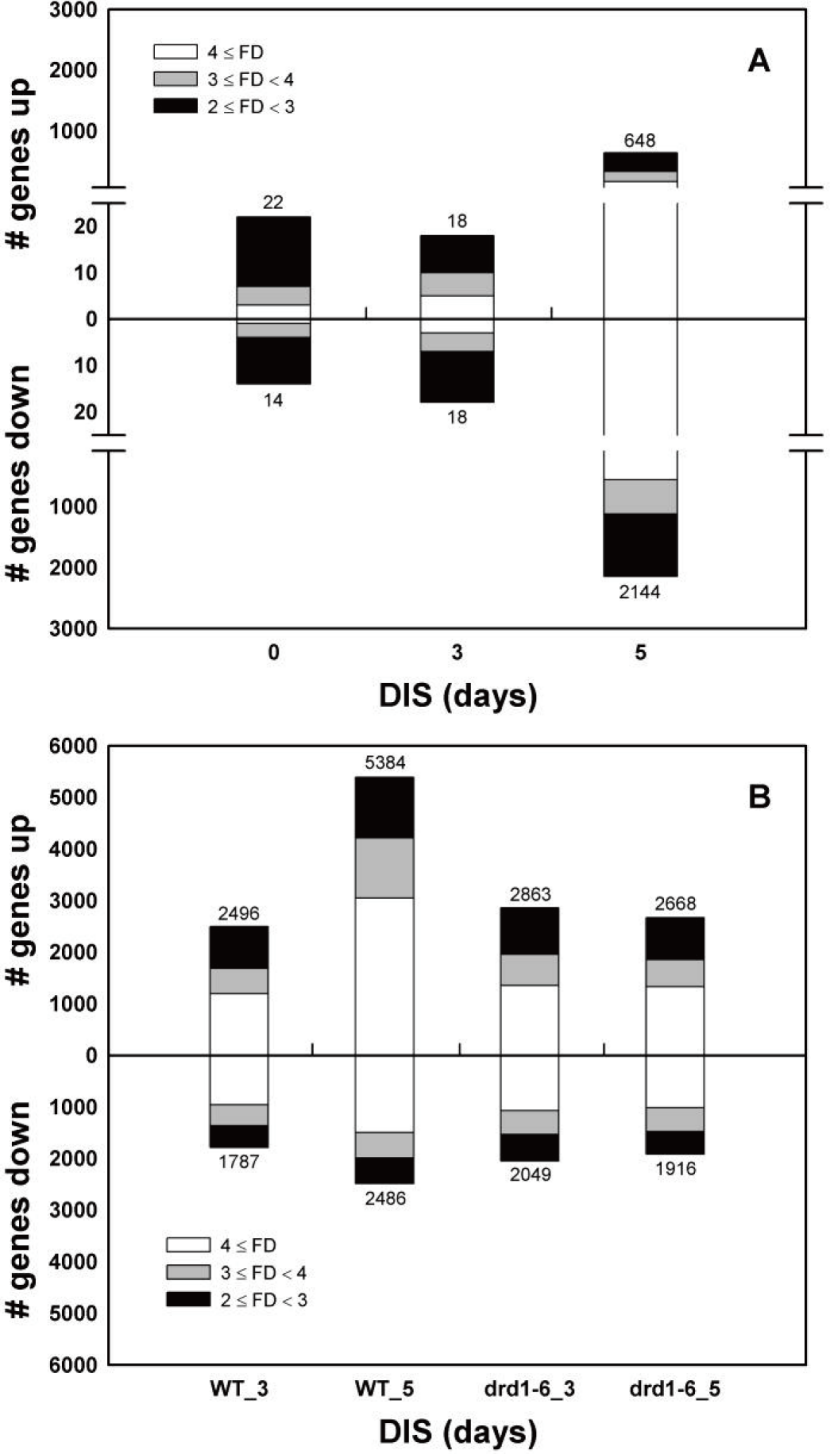
Transcript level changes in rosette leaves during DIS. (A) Microarray analysis represents that the numbers of genes show two- (black), three- (gray), or fourfold (white) up- (upper) or down-(lower) regulation in expression of 0 d, 3 d, and 5 d DIS *drd1-6* mutant compared to WT. (B) The number of genes with two-, three-, or fourfold increase or decrease in gene expression during 3 d and 5 d DIS compared to control (0 d) in the WT and the *drd1-6* mutant are represented by black, gray, and white bars, respectively. Data represent the means of two independent Affymetrix Gene Chip analyses. FD, fold difference.

To reveal differences in gene expression and signaling pathways between WT and *drd1-6* mutant plants during DIS, we compared genome-wide transcriptomes by using the Affymetrix ATH1 Genome Array. The numbers of differentially expressed genes (DEGs) between the WT and the *drd1-6* mutants steeply increased at 5 d of DIS (Fig 6A). As shown in Fig 6B, 5384 genes were upregulated and 2486 genes were downregulated in WT during 5-d DIS, whereas 2668 and 1916 genes were upregulated or downregulated in the *drd1-6* mutants. During DIS, the expression of a higher number of genes changes in the WT than in the *drd1-6* mutants. We identified that 648 and 2,144 genes were upregulated or downregulated in the *drd1-6* mutant relative to WT at 5 d of DIS (Fig 6A). Moreover, gene ontology (GO) analysis of the DEGs showed changes in the primary transcriptome of induced or repressed genes, which were selected for the top 5 significance values based on P values in each GO category at 5-d DIS (Table 1). In the biological process of GO category, the top 5 significance values among the induced genes were most related to photosynthesis. In contrast, the repressed genes were those associated with protein transport, protein localization, vesicle-mediated transport, and autophagy. These results indicate that photosynthetic activity was rapidly reduced in WT. Accordingly, the common 2792 DEGs at 5-d DIS from 2 independent genechip analyses were further analyzed using photosynthesis-related keywords such as chlorophyll, chloroplast, photosystem, or photomorphogenesis. As expected from Fig 4, gene expression levels of many thylakoid structural components constituting photosystems were considerably maintained in the *drd1-6* mutant until 5 d of DIS, whereas those of some proteases were repressed (Table 2).

**Table 1:** Gene ontology analysis of differentially expressed genes (DEGs) between the WT and the *drd1-6* after 5-d DIS.

GOC	GO_ID	GO term	C	*P* value	k
*Induced genes*					
BP	0015979	photosynthesis	175/13998 (1.25)	7.43e-20	37/648 (5.71)
	0006412	translation	1231/13998 (8.79)	3.19e-16	94/648 (14.51)
	0019684	photosynthesis, light reaction	85/13998 (0.61)	1.03e-11	20/648 (3.09)
	0009765	photosynthesis, light harvesting	32/13998 (0.23)	1.69e-09	12/648 (1.85)
	0044271	nitrogen compound biosynthetic process	506/13998 (3.61)	3.43e-06	37/648 (5.71)
CC	0005840	ribosome	470/13779 (3.41)	2.32e-54	101/648 (15.59)
	0030529	ribonucleoprotein complex	671/13779 (4.87)	1.07e-50	114/648 (17.59)
	0043228	non-membrane-bounded organelle	1144/13779 (8.30)	1.29e-48	144/648 (22.22)
	0043232	intracellular non-membrane-bounded organelle	1144/13779 (8.30)	1.29e-48	144/648 (22.22)
	0022626	cytosolic ribosome	317/13779 (2.30)	6.13e-15	77/648 (11.88)
MF	0003735	structural constituent of ribosome	394/14806 (2.66)	2.46e-54	90/648 (13.89)
	0005198	structural molecule activity	583/14806 (3.63)	4.27e-47	95/648 (14.66)
	0019843	rRNA binding	76/14806 (0.51)	4.47e-20	26/648 (4.01)
	0008266	poly(U) RNA binding	20/14806 (0.14)	9.87e-07	8/648 (1.23)
	0008187	poly-pyrimidine tract binding	20/14806 (0.14)	9.87e-07	8/648 (1.23)
*Repressed genes*					
BP	0015031	protein transport	488/13998 (3.49)	5.70e-15	96/2144 (4.48)
	0045184	establishment of protein localization	488/13998 (3.49)	5.70e-15	96/2144 (4.48)
	0008104	protein localization	505/13998 (3.61)	5.18e-14	96/2144 (4.48)
	0016192	vesicle-mediated transport	290/13998 (2.07)	1.83e-12	64/2144 (2.99)
	0006914	autophagy	21/13998 (0.15)	2.52e-12	16/2144 (0.75)
CC	0042579	microbody	177/13779 (1.28)	2.31e-10	43/2144 (2.01)
	0005777	peroxisome	177/13779 (1.28)	2.31e-10	43/2144 (2.01)
	0005788	endoplasmic reticulum lumen	23/13779 (0.17)	5.79e-06	11/2144 (0.51)
	0044432	endoplasmic reticulum part	92/13779 (0.67)	1.38e-05	22/2144 (1.03)
	0031224	intrinsic to membrane	2658/13779 (19.29)	8.90e-05	269/2144 (12.55)
MF	0016791	phosphatase activity	308/14806 (2.08)	3.75e-05	50/2144 (2.33)
	0016701	oxidoreductase activity	72/14806 (0.49)	1.38e-04	18/2144 (0.84)
	0005509	calcium ion binding	405/14806 (2.74)	1.64e-04	59/2144 (2.75)
	0008092	cytoskeletal protein binding	112/14806 (0.76)	2.83e-04	23/2144 (1.07)
	0004721	phosphoprotein phosphatase activity	206/14806 (1.39)	5.82e-04	34/2144 (1.59)

The DEGs were selected to have more than a twofold difference in the *drd1-6* relative to the WT. The 2792 DEGs commonly selected from two independent genechip analyses were used for gene ontology analysis. GOC, BP, CC and MF represent the GO category, biological process, cellular component, and molecular function. GO terms in the table were selected for the top five significances based on *P* values in each GO category. C, the total frequency of a given GO term in all annotations in the *Arabidopsis* genome; k, cluster frequency of a given GO term in the differentially expressed genes selected. Numbers in parentheses are percentages.

**Table 2:** Gene lists closely associated with the phenotypic differences between the WT and the *drd1-6* by DIS.

ID	FD	Description
*Induced genes*		
At5g54270	10.7739395	Lhcb3 chlorophyll a/b binding protein
At2g05070	7.02751945	putative chlorophyll a/b binding protein
At3g08940	6.8425835	putative chlorophyll a/b-binding protein similar to CP29
At4g05180	6.22219935	photosystem II oxygen-evolving enhancer protein 3 precursor—like protein
At1g31330	5.864707	photosystem I subunit III precursor
At4g10340	5.7859877	chlorophyll a/b-binding protein similar to CP26
At3g18490	5.63445015	putative chloroplast nucleoid DNA-binding protein
At1g15820	5.586536	chlorophyll binding protein
At4g02770	5.09564285	putative photosystem I reaction center subunit II precursor similar to PSI-D
At1g74470	4.7775743	geranylgeranyl reductase involving in chlorophyll pathway
At1g55670	4.4582806	photosystem I subunit V precursor
At3g47470	4.1839614	chlorophyll a-b binding protein 4 precursor homolog
At1g67740	3.9063101	putative photosystem II core complex
At2g34430	3.6510979	putative photosystem II type I chlorophyll a b binding protein
At3g51820	3.5500011	chlorophyll synthetase
At1g35680	3.50588475	chloroplast 50S ribosomal protein L21 precursor
At5g35630	3.3689262	chloroplast glutamate-ammonia ligase precursor
At3g54890	3.2839881	chlorophyll a/b-binding protein
At3g16140	3.23015765	photosystem I subunit VI precursor
At4g18480	3.18219105	chloroplast protein ch-42 precursor
At3g50820	3.03489055	putative protein 1 photosystem II oxygen-evolving complex
At1g52230	2.94588435	photosystem I subunit VI precursor
At4g27440	2.84899315	protochlorophyllide reductase precursor
At1g03130	2.7013172	putative photosystem I reaction center subunit II precursor
At3g15000	2.6441301	unknown protein similar to DAG protein required for chloroplast differentiation
At1g34000	2.60216525	hypothetical protein contains similarity to photosystem II 22 kDa protein
At1g61520	2.46207155	PSI type III chlorophyll a/b-binding protein
At4g21280	2.3480178	photosystem II oxygen-evolving complex protein 3
At2g24090	2.2699696	putative chloroplast ribosomal protein L35
At5g09420	2.1607257	putative subunit of TOC complex chloroplast gene Toc64
At3g25920	2.09885045	chloroplast 50S ribosomal protein L15 precursor
*Repressed genes*		
At4g11110	0.45944909	photomorphogenesis repressor COP1 like protein
At5g20300	0.39383131	putative protein chloroplast outer envelope protein OEP86 precursor
At1g06870	0.37378715	chloroplast thylakoidal processing peptidase
At4g12060	0.36089346	putative nuclear gene encoding chloroplast protein CLPC
At4g03320	0.31882931	putative chloroplast protein import component similar to Tic20
At3g59080	0.300814865	putative chloroplast nucleoid DNA binding protein CND41
At5g52250	0.273200955	putative protein similar to photomorphogenesis repressor COP1
At2g17760	0.21195865	putative chloroplast nucleoid DNA-binding protein similar to peptidase family A1

The lists were sorted out from the common 2792 DEGs of two independent genechip analyses by applying keywords as follows: chlorophyll, chloroplast, photosystem, or photomorphogenesis. FD, an average of fold difference for each gene between the WT and the *drd1-6* after 5-d DIS.

### Difference in the epigenetic regulation of senescence between WT and *drd1-6* mutant plants

**Fig 7:**
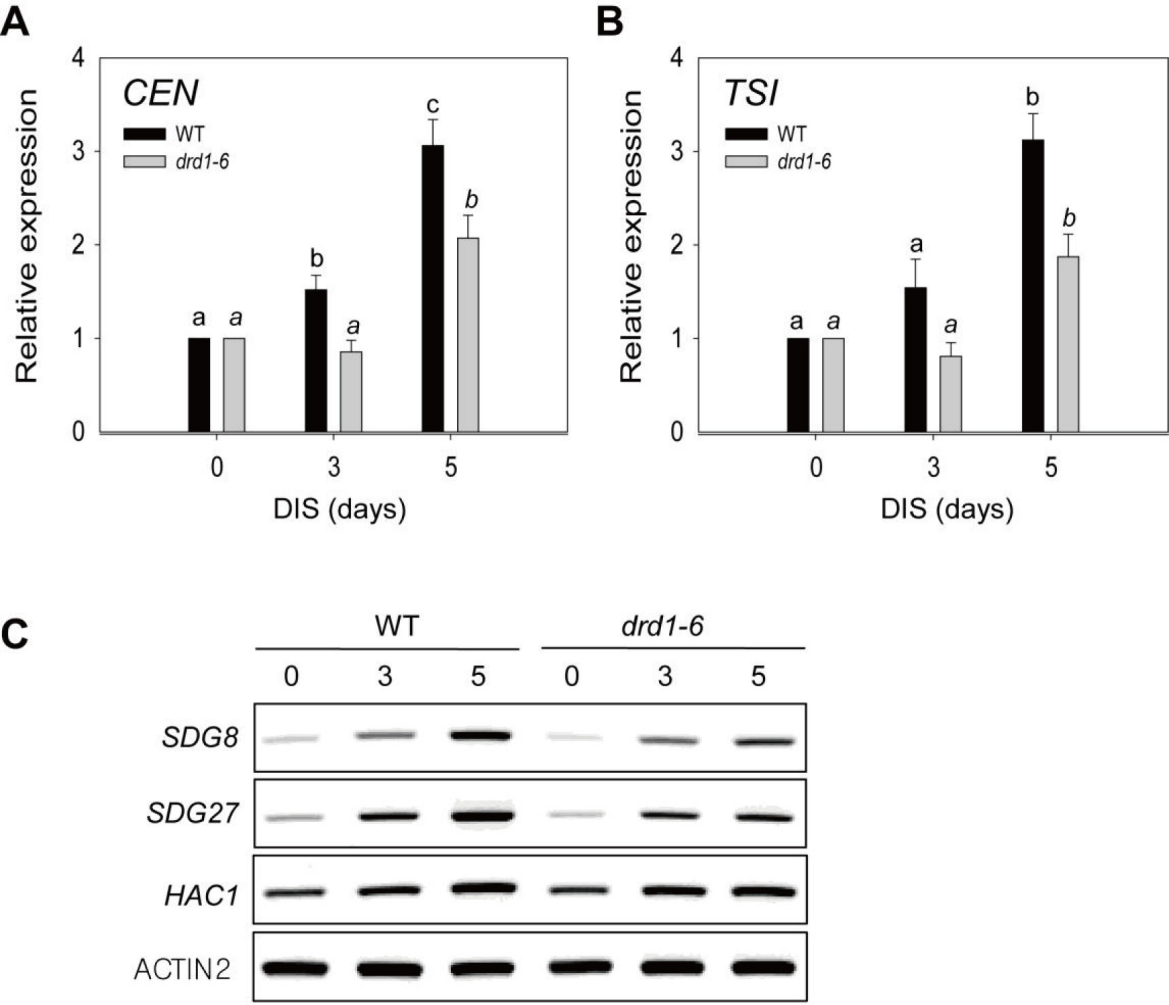
Expression of transcriptional gene silencing markers and histone methyltransferase/acetyltransferase genes in the *drd1-6* mutant. (A) Expression analysis using transcriptional gene silencing markers 180-bp centromeric repeats (*CEN*) and (B) transcriptionally silent information (*TSI*) of WT and the *drd1-6* mutant during DIS. Relative RNA levels were measured by qRT-PCR and the values are normalized to *ACTIN2* expression. Data indicate the mean ± SD (n = 9) from three independent experiments. Bars with the same letter are not significantly different at *P* < 0.05 by Tukey’s honestly significant difference (HSD) test. (C) RT-PCR analysis in the WT and the *drd1-6* mutant leaves at the indicated days. *SDG8*, set domain group 8; *SDG27*, set domain protein 27; and *HAC1*: histone acetyltransferase of the CBP family 1. *ACTIN2* was used as control. Representative data from three or more independent experiments with similar results are shown.

DRD1 is known to participate in RNA-directed DNA methylation [15]. Therefore, to account for the delayed senescence of the *drd1-6* mutant, we compared changes in the epigenetic regulation during DIS between the WT and the *drd1-6* mutant plants via quantitative gene expression analysis of 180-bp centromeric (*CEN*) repeats and pericentromeric repeats termed *TSI* (transcriptionally silent information). The expression levels of *CEN* and *TSI* repeats significantly increased in both the WT and the *drd1-6* mutants during DIS; however, these increases were slower and lesser in the *drd1-6* mutant than in the WT plant (Fig 7A and 7B; S3 Fig). This result suggests that transcriptional gene silencing of *CEN* and *TSI* repeats, which is mediated by DNA methylation, should be released during DIS and this release be slower in the *drd1-6* mutant than in the WT. Moreover, the expression of histone methyltransferase or acetyltransferase genes, such as *SDG8*, *SDG27*, or *HAC1*, exhibited slightly delayed induction with DIS in the *drd1-6* mutant compared to WT, albeit to different degrees (Fig 7C).

### Delayed leaf senescence is shown in *ddm1-2* mutant as well as *drd1-6* mutant

**Fig 8:**
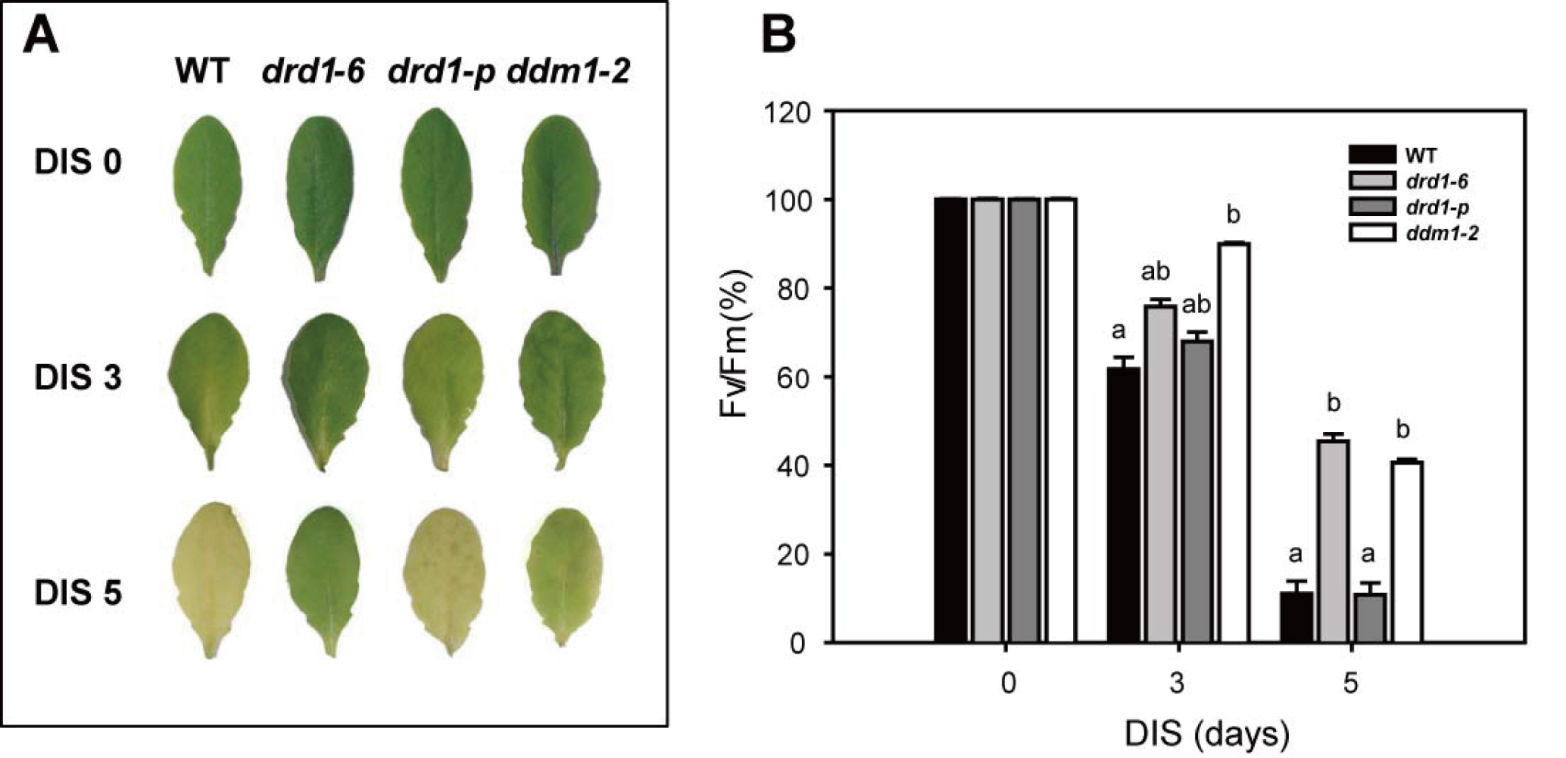
Delayed leaf senescence symptoms in the *ddm1-2* as well as in the *drd1-6* mutant. (A) Phenotypes of detached WT, *drd1-6*, *drd1-p* and *ddm1-2* leaves after 0, 3, and 5-d dark incubation. (B) Photochemical efficiency of photosystem II (Fv/Fm) in WT and mutant leaves in (A). Data represent average values ± SE (n = 27) of three independent experiments. Bars with the same letter are not significantly different at *P* < 0.05 by Tukey’s honestly significant difference (HSD) test.

To support the involvement of DRD1 in leaf senescence, we later obtained another mutant allele of *DRD1*, *drd1-p*, and identified it as a knockdown mutant (Fig 1). When the rosette leaves of the 28-day-old WT, *drd1-6*, and *drd1-p* mutant plants were detached and incubated under darkness for 5 days, the *DRD1* knockdown mutant, *drd1-p*, did not exhibit a noticeable phenotype of delayed senescence compared to WT (Fig 8). In addition, we observed no difference between the WT and *drd1-p* mutant in vegetative development and flowering time. Although the expression of *DRD1* gene was downregulated in the *drd1-p* mutant, the putative role of DRD1 in regulation of leaf senescence as well as development seems to be not affected in this mutant. Some DRD1 proteins might be produced enough to regulate leaf senescence in the *drd1-p* mutant. Therefore, we hypothesized that the helicase superfamily C-terminal (HELICc) domain of SWI2/SNF2 chromatin remodelers is important for regulation of leaf senescence. To test this hypothesis, we investigated DIS in another mutant, *ddm1-2*, which has a mutation in the helicase domain of DDM1, a SWI2/SNF2-like chromatin remodeling protein like DRD1. The substitution of G to A in the *ddm1-2* mutant is predicted to disrupt the HELICc domain because of premature translation termination (Fig 1). Interestingly, the *ddm1-2* mutant leaves stayed green and maintained structural integrity for a longer period than the wild-type leaves did during DIS (Fig 8A). Similar to the *drd1-6* mutant, this mutant also exhibited delayed leaf senescence in terms of the photochemical efficiency, Fv/Fm. The photochemical efficiency dropped to 61.7% or 89.9% of the control in the wild-type or the *ddm1-2* leaves at 3-d of DIS and then to 11.0% or 40.6% at 5-d of DIS, respectively (Fig 8B). These data suggest that both DRD1 and DDM1 may influence leaf senescence, and that the helicase domain of these SWI2/SNF2-like chromatin remodeling proteins might be important for regulation of leaf senescence.

## Discussion

Leaf senescence is a complex process in which the effects of diverse factors are integrated into complicated genetic regulatory networks involving extensive transcriptional control [6, 7]. In this study, we showed a significantly delayed leaf senescence phenotype in the *drd1-6* mutant, confirmed by changes in senescence parameters, including chlorophyll and protein content, the maximum photochemical efficiency (Fv/Fm), and the maximal electron transport rate (ETRmax) (Figs 2–4). Leaf senescence is a genetically regulated developmental process that leads to cell death [2]. Chlorophyll degradation, which is an active process to salvage nutrients from old tissues, is the first symptom of leaf senescence [3, 7]. The chlorophyll pathway has previously been examined [34] and numerous genes in the pathway have been studied. The key enzyme of this pathway appears to be the one encoding pheophorbide α oxygenase (PAO), which cleaves the tetrapyrrole ring to produce red chlorophyll catabolite (RCC) [35]. The expression of *PAO* gene dramatically increased during senescence (Fig 5) in the WT plants, implicating this enzyme as a control point in the process [7]. Moreover, chlorophyll breakdown is tightly connected with the removal of pigment-protein complexes and the degradation of chlorophyll-binding protein [31]. The first step of chlorophyll degradation is the conversion of chlorophyll b in the light-harvesting complex II (LHCII) trimer to 7-hydroxymethyl chlorophyll a, in a process that is catalyzed by chlorophyll b reductase (CBR) [36]. As shown in Fig 4, LHCII almost disappeared after 5-d dark incubation in WT, and *CBR* gene was highly expressed in WT plants (Fig 5). The D1 protein, responsible for degradation of chlorophyll-binding proteins has a rapid turnover rate not only under photoinhibitory conditions, but also during senescence [37]. The photosynthetic complex structure of the thylakoid membrane proteins in chloroplast remained and did not change dramatically under senescence induction in the *drd1-6* mutants (Fig 4), suggesting that the leaf senescence of the *drd1-6* mutant is significantly delayed.

A massive decrease in RNA and protein synthesis as well as in the total levels of these constituents occurs during leaf senescence [38]. The most abundant chloroplast protein is ribulose-1,5-bis-phosphate carboxylase/oxygenase (Rubisco), whose levels decrease rapidly in the early phase of leaf senescence [39], although the mechanism of intrachloroplastic Rubisco degradation is still unknown [40]. In total protein levels, Rubisco was gradually degraded in the WT during DIS, but not in the *drd1-6* mutant (Fig 4). As shown in Fig 4, total protein was rapidly degraded in the WT, indicating that DIS occurs faster in the WT plants than in the *drd1-6* mutant.

Leaf senescence involves the induction of a genome-wide redirection of gene expression [1, 7, 41, 42]. Molecular genetic analyses identified transcript levels of senescence-associated genes, *SAG12* and *CBR*, specifically upregulated in senescing leaves (Fig 5). To identify genes that are upregulated or downregulated during leaf senescence in *Arabidopsis* [6, 41], we performed Affymetrix GeneChip microarray. Microarray data can be used as a tool to develop and test hypotheses for transcriptional control during senescence [43]. In senescing leaves, many of the genes, e.g., those encoding photosynthetic proteins, are generally downregulated, whereas other senescence-associated genes (*SAGs*) are upregulated. According to our microarray results, such expression changes were much more conspicuous in the WT compared with the *drd1-6* mutants (Fig 6B). Moreover, GO analysis of DEGs showed that a large number of genes participating in photosynthesis were substantially downregulated in the WT plants (Tables 1 and 2).

DRD1 is a member of the plant-specific subfamily of SWI2/SNF2 chromatin remodeling proteins. Although SWI2/SNF2 chromatin remodeling proteins play roles in plant development and stress response [44–46], their roles in leaf senescence remain unknown. In this study, we showed that *DRD1* mutation delayed leaf senescence substantially based on various senescence parameters. As shown in Figs 2 and 4, the effects of *DRD1* mutation were increasingly manifested with progress of leaf senescence. Moreover, the *drd1-6* mutant exhibited not only a delay in leaf senescence, but also a prolonged whole life span. Although it was reported that a mutation in *DRD1* gene did not affect flowering time [27], the delayed flowering phenotype was observed in the *drd1-6* mutant with a several-day variation compared to the WT plants. Accordingly, *DRD1* mutation seems to bring pleiotropic effects on development including flowering and leaf senescence. Since the effects of *DRD1* mutation on leaf senescence can be attributed to the delayed development, we substantiated the delayed leaf senescence in the *drd1-6* mutant by comparing DIS of the 28-day-old WT and 34, 36, and 38-day-old *drd1-6* plants (Fig 3). This result indicates that DRD1 is substantially involved in leaf senescence as well as plant development. It is also worthy to note that the delayed leaf senescence was observed in the *ddm1-2* mutant as well as the *drd1-6* mutant (Fig 8). In contrast, the *drd1-p* mutant, in which *DRD1* gene was downregulated, did not show such a phenotype. This normal phenotype in the *drd1-p* mutant is possibly attributed to the existence of some intact DRD1 proteins, which is expected by the lower transcription level. However, as described above, *DDM1* and *DRD1* genes are grouped into the same SWI2/SNF2 family of ATP-dependent chromatin remodelers and both the *ddm1-2* and *drd1-6* mutants have a mutation in the helicase domain. Taken together, these results suggest that the ATP-helicase domain of SWI2/SNF2 chromatin remodelers may be probably important for regulation of leaf senescence.

An age-associated decline in total genomic DNA methylation occurs [12, 47, 48]. Plant DNA can trigger both symmetric (CpG and CpNpG) and asymmetric DNA methylation (CpNpN) [49], which are associated with repressive chromatin gene promoters and with repression of gene expression [13]. Most DNA methylation occurs in transposon-rich heterochromatic regions [50, 51] and leads to transcriptional gene silencing (TGS). TGS openness inactivates foreign genes integrated into plant genomes but likely also suppresses an unknown subset of chromosomal information [52]. The *drd1-6* mutant expression is suppressed by endogenous repeats such as *CEN* and *TSI* (Fig 7A and 7B). Therefore, one of possible mechanisms underlying the epigenetic regulation of leaf senescence is that DRD1 may regulate leaf senescence positively via RNA-directed DNA methylation (RdDM) of endogenous repeats. Actually, it has been reported that activation of transposable elements during senescence and senescence-triggering stresses could affect the expression of neighboring genes [53, 54]. Environmental factors such as salt and pathogen also bring about change in DNA methylation, leading to altered transcription of repetitive sequences and/or neighboring genes [55, 56].

Another possibility is chromatin remodeling-mediated control of senescence-associated gene expression. Similar to ORE7, DRD1 may induce changes of chromatin structures directly. Then, the induced changes of chromatin structures by DRD1 can allow transcription proteins to access permissive chromatin. Otherwise, DRD1 may lead to alteration of chromatin structure via altered histone modification indirectly. SDG is an HKMT (histone lysine methyltransferase), a chromatin-modifying enzyme [57], and HAC1 is a histone acetyltransferase (HAT) belonging to the CBP family. *SDG8*, *SDG27*, and *HAC1* are expressed at low levels in the *drd1-6* mutant compared with WT plants (Fig 7C). Consequently, senescent WT plants are expected to show greater histone methylation or acetylation activity. The interphase chromatin of senescing leaves shows a traditional partitioning of histone modification marks indexing euchromatic and heterochromatic domains [11]. During leaf senescence, global changes in chromatin organization correlate with the massive changes in the transcriptome observed, including downregulation of numerous genes and induction of many senescence-associated genes and photosynthesis-related genes (Tables 1 and 2) [7, 41, 58]. In conclusion, these results indicate that such epigenetic modifications may contribute to replicative senescence, and a mutation in DRD1 gene can influence the progress of leaf senescence. However, the exact underlying mechanisms by which DRD1 is involved in leaf senescence, remain to be further elucidated.

## Supporting Information

S1 Fig Phenotype of detached WT and *drd1-6* mutant leaves at the indicated days during DIS. (JPG)

S2 Fig SDS-PAGE analysis in WT and *drd1-6* mutant during DIS. Twenty micrograms of total protein was isolated from WT and *drd1-6* mutant rosette leaves at the indicated days. Total proteins were loaded 12% SDS-PAGE gel and stained by coomassie blue.
(JPG)

S3 Fig Relative gene expression of *CEN* and *TSI* during DIS. (A) Expression levels of transcriptional gene silencing markers 180-bp centromeric repeats and (B) *TSI* in *drd1-6* mutant, relative to those in WT during DIS. Relative transcript levels were measured by quantitative real-time PCR and the values are normalized to *ACTIN2* expression. Data indicate the mean ± SD (*n* = 9) from three independent experiments.
(JPG)

S1 Table Primer lists used in this study. (DOCX)

S2 Table Identification of thylakoid membrane proteins which are declined during DIS in *Arabidopsis*. GI, GenInfo Identifier in the NCBI database; Mass, predicted molecular mass; PI, calculated isoelectric point; and % coverage, protein sequence coverage.
(DOCX)
