# Supplementary figures and images for: A Mutation in Plant-Specific SWI2/SNF2-Like Chromatin-Remodeling Proteins, DRD1 and DDM1, Delays Leaf Senescence in Arabidopsis thaliana
Source: PLoS One. 2016 Jan 11;11(1):e0146826. doi: 10.1371/journal.pone.0146826 (PMC4709239; doi:10.1371/journal.pone.0146826)

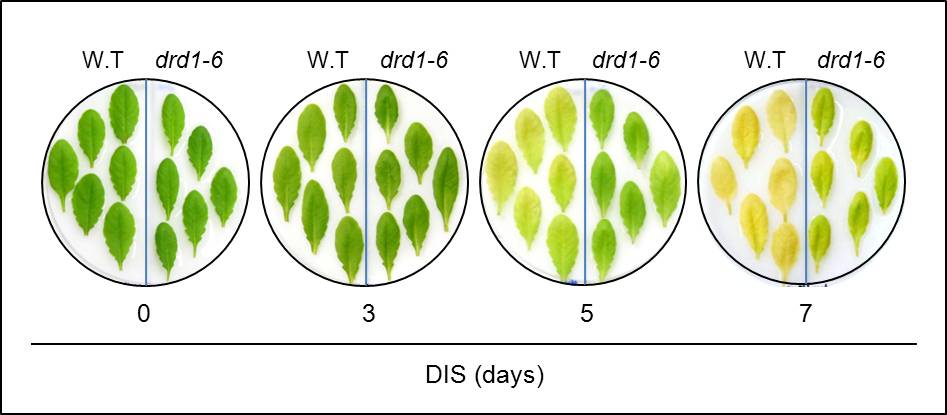

Supplement: S1 Fig — (JPG) [file pone.0146826.s001.jpg]

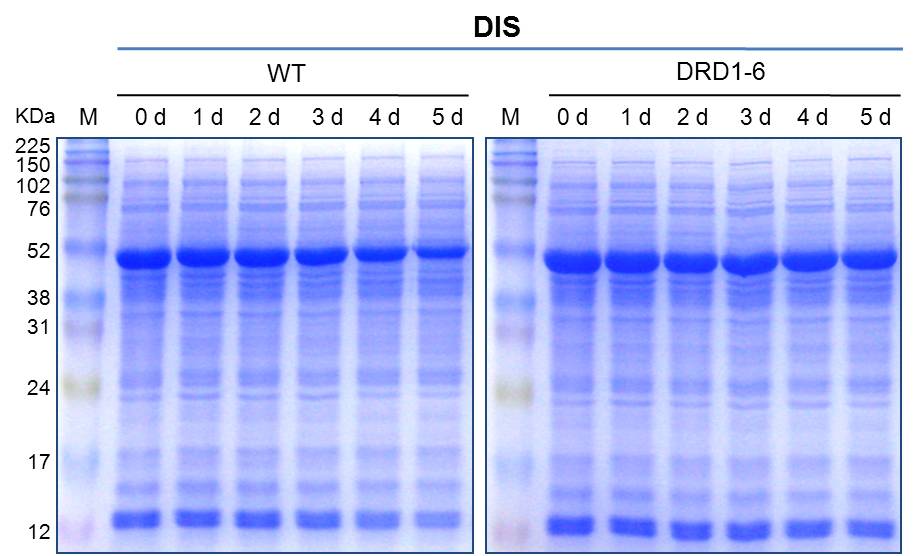

Supplement: S2 Fig — Twenty micrograms of total protein was isolated from WT and drd1-6 mutant rosette leaves at the indicated days. Total proteins were loaded 12% SDS-PAGE gel and stained by coomassie blue. (JPG) [file pone.0146826.s002.jpg]

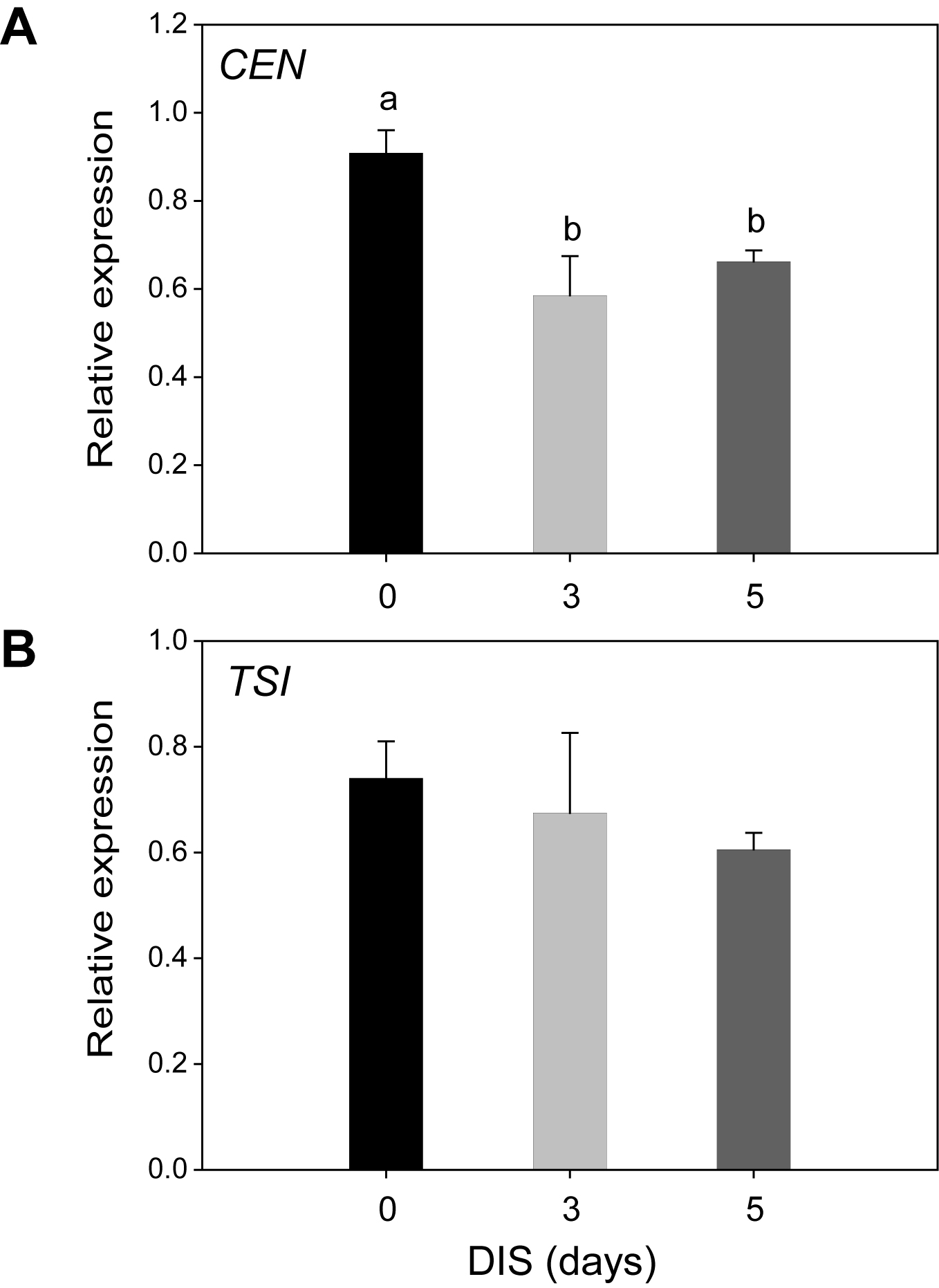

Supplement: S3 Fig — (A) Expression levels of transcriptional gene silencing markers 180-bp centromeric repeats and (B) TSI in drd1-6 mutant, relative to those in WT during DIS. Relative transcript levels were measured by quantitative real-time PCR and the values are normalized to ACTIN2 expression. Data indicate the mean ± SD (n = 9) from three independent experiments. (JPG) [file pone.0146826.s003.jpg]
