# Supplementary material for: A Mutation in Plant-Specific SWI2/SNF2-Like Chromatin-Remodeling Proteins, DRD1 and DDM1, Delays Leaf Senescence in Arabidopsis thaliana
Source: PLoS One. 2016 Jan 11;11(1):e0146826. doi: 10.1371/journal.pone.0146826 (PMC4709239; doi:10.1371/journal.pone.0146826)
Supplement: S1 Table — (DOCX) [file pone.0146826.s004.docx]

**S1 Table. Primer lists used in this study.**

| Gene | Sense / antisense primer sequence (5'→3') | Size (bp) |
| --- | --- | --- |
| *SAG12* | GTGACCCCTATCAAGAATCA / CCAGTCGCTTTTATATGCTC | 200 |
| *ANS* | TATGCAAACTCACCGGACAC / GCCTTTCGCACAGTTTTCTC | 205 |
| *CBR* | AATTACCTAACTCCTCCTCGGATATT / GAGAGAAAACAGAGACCCAAGTATTC | 202 |
| *PAO* | ACGGCATGGTAAGAGTCAGC / AAACCAGCAAGAACCAGTCG | 249 |
| *SDG8* | AGAAGCTCCTCAGGCTAAAG / CTTCCTTCCTTACCTCCATC | 204 |
| *SDG27* | GGAAAGGTTGTGACAGACTC / AACTGCTCTCCACTATCTGC | 194 |
| *HAC1*  *DRD1* | CTCGATCTTCTGGTACATGC / GAGGTACATGGCACTCTGAT  CTCCCAAAAGGTATTCTGCCTAC/GGTAAATTGCACTTCCCACAGCC | 199  766 |
| *CEN* | ACCATCAAAGCTTTGAGAAGCAAGAAGAAGCTT /  CCATATGAGTCTTTGTCTTTGTATCTTCT | 167 |
| *TSI* | CTCTACCCTTTGCATTCATGAATCCTT / GATGGGCAAAAGCCCTCGGTTTTAAAATG | 175 |
| *ACTIN2* | GCCCAGAAGTCTTGTTCCA / CTTGGTGCAAGTGCTGTGAT | 199 |
