# Supplementary material for: A Mutation in Plant-Specific SWI2/SNF2-Like Chromatin-Remodeling Proteins, DRD1 and DDM1, Delays Leaf Senescence in Arabidopsis thaliana
Source: PLoS One. 2016 Jan 11;11(1):e0146826. doi: 10.1371/journal.pone.0146826 (PMC4709239; doi:10.1371/journal.pone.0146826)
Supplement: S2 Table — GI, GenInfo Identifier in the NCBI database; Mass, predicted molecular mass; PI, calculated isoelectric point; and % coverage, protein sequence coverage. (DOCX) [file pone.0146826.s005.docx]

**S2 Table. Identification of thylakoid membrane proteins which are declined during DIS in *Arabidopsis*.** GI, GenInfo Identifier in the NCBI database; Mass, predicted molecular mass; PI, calculated isoelectric point; and % coverage, protein sequence coverage.

| **GI** | **Protein name** | **Mass** | **PI** | **% coverage** |
| --- | --- | --- | --- | --- |
| 7525046 | cytochrome f | 35335 | 8.34 | 49 |
| 15230324 | oxygen-evolving enhancer protein 1-2 | 34998 | 5.92 | 53 |
| 7525028 | photosystem II protein D2 | 39522 | 5.46 | 17 |
| 15081739 | light harvesting complex photosystem II (LHCB4.1) | 31192 | 5.76 | 15 |
| 15228268 | chloroplastic lipocalin | 39090 | 6.36 | 5 |
| 18416029 | PGR5-like protein 1A | 35698 | 5.20 | 14 |
| 15235029 | chlorophyll a-b binding protein CP26 | 30138 | 6.00 | 25 |
| 15237201 | calcium sensing receptor | 41260 | 9.42 | 10 |
| 430947 | PSI type III chlorophyll a/b-binding protein | 29085 | 8.61 | 16 |
| 342351105 | Chain A, Crystal Structure Of The Tl29 Protein | 29329 | 7.05 | 13 |
| 7525013 | photosystem II protein D1 | 38911 | 5.12 | 11 |
| 15231990 | chlorophyll a-b binding protein CP29.2 | 31174 | 5.85 | 16 |
| 18415378 | proline-rich family protein | 24380 | 4.72 | 6 |
| 18403751 | putative plastid-lipid-associated protein 6 | 30436 | 5.82 | 16 |
| 15238000 | S-ribonuclease binding protein | 33466 | 6.17 | 7 |
